# Supplementary material for: Identifying Different Mutation Sites Leading to Resistance to the Direct-Acting Antiviral (DAA) Sofosbuvir in Hepatitis C Virus Patients from Egypt
Source: Microorganisms. 2022 Mar 22;10(4):679. doi: 10.3390/microorganisms10040679 (PMC9024585; doi:10.3390/microorganisms10040679)
Supplement: Supplementary file 1 [file microorganisms-10-00679-s001.zip › microorganisms-1629978-supplementary.pdf]

Supporting information

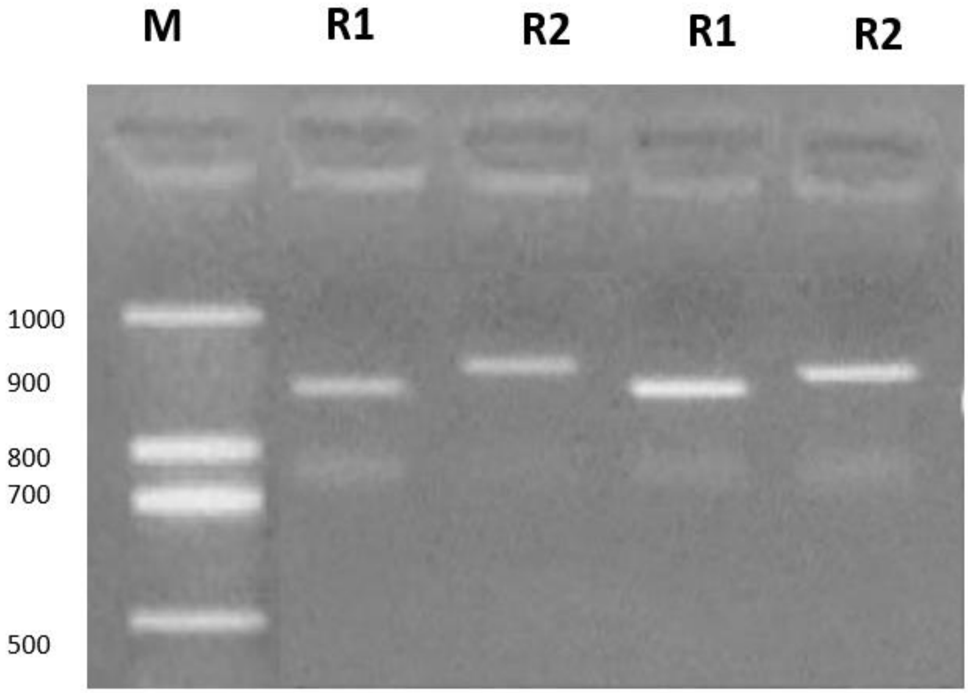

Gel represent sample number one and sample number two with total size around 1800 bp

M: designates the well containing a100 bp DNA ladder

R1: region one with 880 bp

R2: region two with 922 bp

Figure S1. Gel electrophoresis represents sample no.1 and sample no.2 with a total size of around 1800 bp. (R1) Region 1 with size 822bp, and (R2) Region 2 with size 922bp

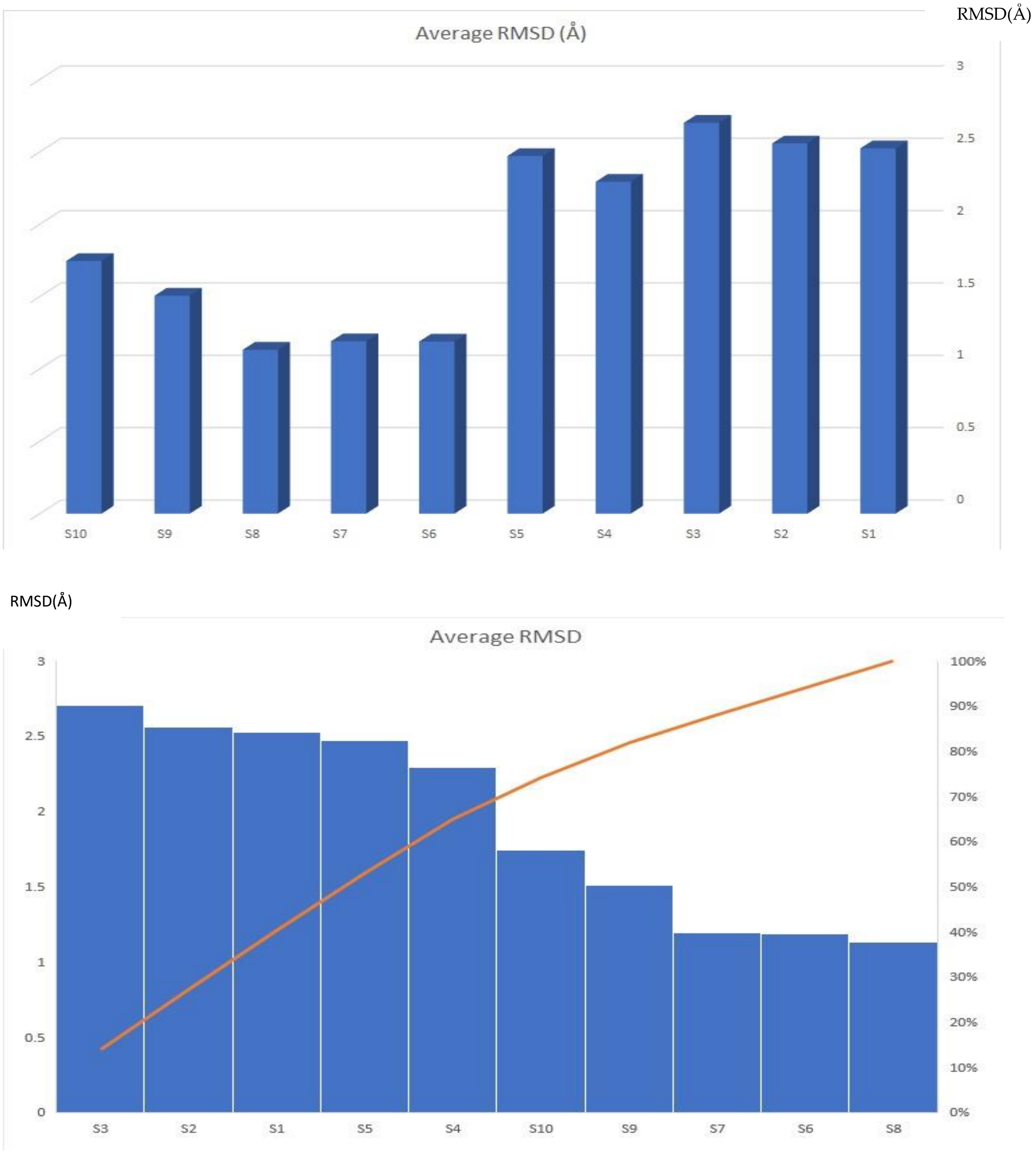

Figure S2. Histogram showing the average RMSD of ten dynamic simulation experiments for HCV 4a NS5B resistant(S1:S5) and responder samples(S6:S10).
